# A previously unknown way of heme detoxification in the digestive tract of cats

**DOI:** 10.1038/s41598-021-87421-6

**Published:** 2021-04-15

**Authors:** Alexandr B. Duzhak, Petr S. Sherin, Vadim V. Yanshole, Sergey L. Veber, Sergey I. Baiborodin, Olga I. Sinitsyna, Yuri P. Tsentalovich

**Affiliations:** 1 Laboratory of Proteomics and Metabolomics, International Tomography Center SB RAS, Institutskaya str. 3a, Novosibirsk, 630090 Russia; 2 Group of Photoinduced Processes, International Tomography Center SB RAS, Institutskaya str. 3a, Novosibirsk, 630090 Russia; 3 Department of Chemistry, Imperial College London, Molecular Sciences Research Hub, 82 Wood Lane, White City Campus, London, W12 0BZ UK; 4 Novosibirsk State University, Pirogova str. 2, Novosibirsk, 630090 Russia; 5 Laboratory of Magnetic Resonance, International Tomography Center SB RAS, Institutskaya str. 3a, Novosibirsk, 630090 Russia; 6 Common Use Centre for Microscopy of Biological Subjects, Institute of Cytology and Genetics SB RAS, Acad. Lavrentiev Avenue 10, Novosibirsk, 630090 Russia; 7 Laboratory of Gene Engineering, Institute of Cytology and Genetics SB RAS, Acad. Lavrentiev Avenue 10, Novosibirsk, 630090 Russia

**Keywords:** Biochemistry, Chemical biology, Gastroenterology

## Abstract

Free heme is a highly toxic molecule for a living organism and its detoxification is a very important process, especially for carnivorous animals. Here we report the discovery of a previously unknown process for neutralizing free heme in the digestive tract of domestic cats. The cornerstone of this process is the encapsulation of heme into carbonated hydroxyapatite nanoparticles, followed by their excretion with faeces. This way of heme neutralization resembles the formation of insoluble heme-containing particles in the digestive tracts of other hematophagous species (for example, the formation of insoluble hemozoin crystals in malaria-causing *Plasmodium* parasites). Our findings suggest that the encapsulation of heme molecules into a hydroxyapatite matrix occurs during the transition from the acidic gastric juice to the small intestine with neutral conditions. The formation of these particles and their efficiency to include heme depends on the bone content in a cat’s diet. In vitro experiments with heme-hydroxyapatite nanoparticles confirm the proposed scenario.

## Introduction

**Figure 1:**
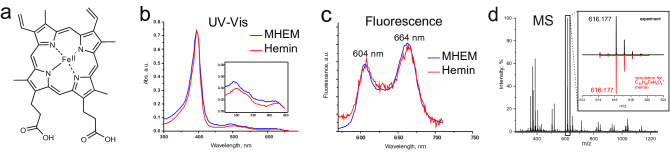
Evidence of heme in MeHCl extracted material (MHEM) of cat faeces. (**a**) Chemical structure of heme (ferriprotoporphyrin IX). (**b**) Absorption spectra of MHEM and hemin solution in MeHCl. MHEM absorption spectrum with Soret band (397 nm) and four Q bands (inset) is very similar to that of hemin. (**c**) Normalized fluorescence spectra of MHEM from cat faeces and hemin in MeHCl, excitation at 397 nm. The fluorescence spectrum of MHEM completely coincides with the spectrum of hemin. (**d**) LC–UV–MS analysis of MHEM. Mass spectrum of peak with m/z 616.177 corresponds to the molecule of heme. Detailed information on the LC–UV–MS analysis of MHEM is presented in Supplementary Fig. S3.

Heme (iron protoporphyrin IX) is a complex organic molecule formed by four pyrrole heterocycles combined into a ring with an iron ion in the centre of the ring (Fig. 1a). Being a part of hemoglobin and myoglobin proteins, heme is one of the key elements of an important biological process—oxygen transfer from the lungs to the tissues. However, in free form the heme molecule can be dangerous for biological tissues. The lipophilic heme molecules can penetrate into the epithelial cells, facilitate the formation of the reactive oxygen species^1,2^, and cause dysfunction of various cellular structures^3,4^.

Normally, free heme comes into the body as a result of natural destruction of erythrocytes, or it can be released from the heme-containing food. There are several mechanisms of free heme detoxification in animals. The most studied mechanism operating in humans and other mammals includes a complex cascade of catabolic reactions of heme oxidation and decomposition to bilirubin and other derivatives followed by the excretion of resulting products with faeces^5,6^. Consumption of food containing high amounts of heme (for example, red meat) may overload this detoxification pathway leading to accumulation of free heme within the body. This remaining heme can have dangerous consequences—from natural intestinal microflora impairment^7^ to colorectal cancer^8^.

Another group of mechanisms of heme detoxification was found in hematophagous organisms including malaria-causing *Plasmodium* parasites^9,10^ and several other blood-feeding invertebrates^11–14^. These mechanisms for heme detoxification can be merged together on the basis of heme aggregation and its deposition in biologically inert form^15^. One predominant form of such aggregates represent heme crystals, called hemozoin^9,16,17^. These crystals are insoluble in physiological fluids and therefore harmless to parasites.

However, a large group of carnivorous creatures (mammals, birds, reptiles, etc.) apparently does not suffer from poisoning by heme despite the diet mainly consisting of meat. It is possible that in addition to the known mechanisms, predators have other ways to neutralize excess amounts of consumed heme to protect themselves from its toxic effects. Considering that many taxonomically different blood-sucking parasites have the same mechanism of heme detoxification through hemozoin formation, one might suspect the existence of a common and currently unknown process of heme detoxification in higher carnivorous organisms.

This article presents such a process and sheds light on its mechanism. We found that cat faeces contain water-insoluble hydroxyapatite nanoparticles with the inclusion of heme molecules. Encapsulation of toxic heme in these particles makes it biologically inert. Our findings suggest that heme-containing particles are formed in chyme during its transition from the stomach to the bowels and excreted from the body as part of faeces.

## Results and discussion

### Search for heme and its derivatives in cat faeces

The objects of our research were carnivorous mammals, namely domestic cats *Felis silvestris catus*. We based the choice of objects on the dual role of cats being typical predators and friendly pets. This provides easy control of food, health status, and the collection of samples for analysis.

We assumed that the products of heme catabolism in faeces of carnivorous mammals, in particular cats, may include the remaining free heme and water-insoluble heme derivatives similar to hemozoin besides the usual heme degradation products (stercobilin and urobilin). To verify this assumption, cats were fed with chicken meat and bones (MB diet) for 3 weeks. Fresh samples of their faeces were collected and then treated according to the protocol below. The extracted fractions were first analysed by UV–Vis spectrometry with subsequent detailed study by fluorimetry, liquid chromatography–UV–mass spectrometry (LC–UV–MS), Fourier transform infrared (FTIR) spectroscopy and transmission electron microscopy (TEM).

1. Extraction of water-soluble inorganic and organic substances (including sterco- and urobilin): 5 mL of water was added to 50 mg of dried faeces; the mixture was shaken in a shaker for 30 min, and then centrifuged (10,000×*g*, 10 min, 20 °C). The supernatant was removed and the pellet was washed twice with water.
2. Extraction of alcohol-soluble substances: methanol was added to the obtained water-insoluble pellet, shaken in a shaker for 30 min, and then centrifuged (10,000×*g*, 10 min, 20 °C). The supernatant was removed, and the procedure was repeated two more times to extract remaining free heme and/or other tetrapyrroles. All three extracts were combined together.
3. Extraction of water- and alcohol-insoluble heme (which we assumed to be a hemozoin-like substance) from the obtained pellet. To find a solvent suitable for dissolution of water- and alcohol-insoluble heme, preliminary experiments were performed with β-hematin, a well-known synthetic analogue of hemozoin^18,19^. β-Hematin was synthesized as previously described^20^ and its identity was confirmed by FTIR spectrum typical for hemozoin^20–22^ (Supplementary Fig. S1a). We found that β-hematin is almost insoluble in a broad range of organic solvents, including alcohols (methanol, ethanol, 1-propanol, 2-propanol, butanol), simple alkanes (hexane, heptane), chloroform, acetonitrile and dimethyl sulfoxide but it could be completely dissolved in aqueous solutions with 50 mM NaOH and some acidified alcohols (in particular, methanol, ethanol, and 1-propanol containing 25 mM HCl) that were prepared shortly before use. Acidified methanol (MeHCl) was chosen as the most suitable solvent for further experiments. The absorption spectrum of resulting β-hematin solution in methanol with the addition of 25 mM HCl was identical to the hemin (commercial chloroferriprotoporphyrin IX) spectrum in the corresponding solvent (Supplementary Fig. S1b,c) confirming the complete dissolution of β-hematin.

Analysis of cat faeces demonstrated (Supplementary Fig. S2) that both water and methanol soluble fractions contained small amounts of free heme and heme degradation products (urobilin and others). Heme extractions from the water- and alcohol-insoluble pellet by 50 mM NaOH were unsuccessful: only traces of urobilin and some unrecognized compounds were found in the solution. This implicitly pointed to the absence of expected hemozoin in cat faeces. However, it was found that the material extracted in MeHCl (MHEM) has the absorption spectrum with the intense Soret band (397 nm) and four significantly smaller Q bands (450–650 nm) which is very similar to the spectrum of hemin (Fig. 1b). The slight mismatch in the Q band position of MHEM and hemin (Fig. 1b, inset) is most likely due to the presence of urobilin admixtures in MHEM. It should be noted that the shapes of Q-bands are highly sensitive to the heme core modification^23^ and the similarity of absorption spectra in Q band region indicates the presence of heme in the obtained extract. The latter is additionally supported by the coincidence of fluorescence spectra of MHEM and hemin (Fig. 1c).

MHEM was also subjected to LC–UV–MS analysis (Fig. 1d; Supplementary Fig. S3). We found that the extracted heme eluted with the same retention time (RT) of hemin and β-hematin chemical standards (RT = 77 s), and it had a major MS peak with m/z 616.177 corresponding to the molecule of protoporphyrin containing Fe ion (C_34_H_32_FeN_4_O_4_^+^, theoretical m/z 616.177) that is firmly confirmed by the isotopic distribution typical for iron. MHEM also contains urobilin found at RT = 59 s, with m/z 591.318 and UV_max_ at 491 nm (C_33_H_43_N_4_O_6_^+^, theoretical m/z 591.318).

Thus, the combination of UV–Vis absorption, fluorescence, and LC–UV–MS data unambiguously demonstrates the presence of heme in MHEM from cat faeces (Fig. 1). The neutralization of MHEM with an aliquot of NaOH led to the immediate precipitation forming an amorphous brownish material. The precipitate as well as the initial MHEM was insoluble in water, methanol, and 50 mM NaOH, but it was completely dissolved in MeHCl with the full recovery of an optical spectrum of the solution similar to that of heme.

Small amounts of urobilin and free heme in water and methanol extracts of faeces (Supplementary Fig. S2) indicate that only a small fraction of heme released from the cat food (MB diet) is utilized via the mechanisms leading to urobilin formation. This may indicate that inside the gastrointestinal tract, heme is protected from enzymes and microorganisms by a structural matrix. This hypothesis was supported by a control experiment, where we revealed the presence of heme in MHEM of dried faeces even after several years of storage at 4–8 °C. Our subsequent efforts were aimed at understanding the nature of this matrix.

### FTIR experiments with heme-protecting matrix

**Figure 2:**
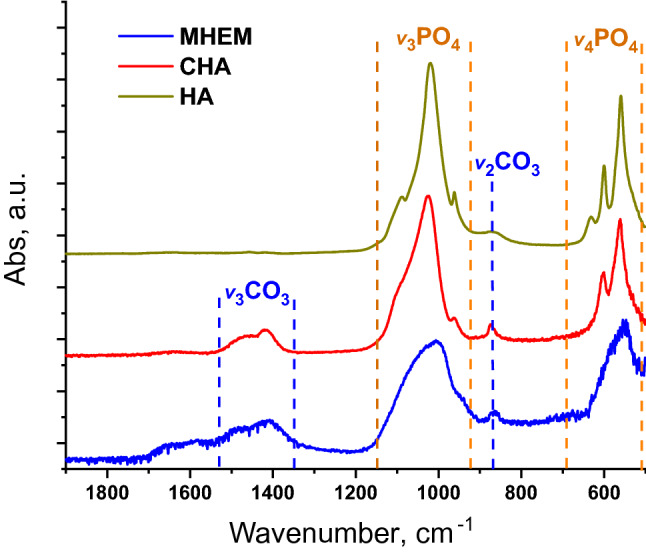
FTIR spectra of control samples of commercial hydroxyapatite (HA), synthetic carbonated hydroxyapatite (CHA), and test sample of precipitated particles of MHEM from cat faeces (MB diet). It is clear that MHEM spectrum presents absorbance bands of orthophosphate and carbonate components specific for hydroxyapatite and in particular for CHA^24–30^.

Molecular components of complex substances can be identified by IR spectroscopy due to well-defined absorption bands of specific chemical bonds. The FTIR spectrum of the MHEM precipitate (Fig. 2) contains two groups of peaks corresponding to orthophosphates and carbonates similar to well-known FTIR spectra of carbonated hydroxyapatites (CHA)^24–30^. The only difference is a smoother shape of phosphate bands in MHEM as compared with standard CHA. This difference might possibly originate from the short time interval between MHEM precipitation and its analysis and, as a consequence, amorphous structure of CHA due to its immaturity^30^.

Hydroxyapatite (Ca_10_(PO_4_)_6_(OH)_2_) is a mineral widespread in nature. Biogenic hydroxyapatites with different level of carbonization are the major inorganic components of bones and teeth. They can be formed from calcium orthophosphates and carbonates present in biological fluids^28,30^. The addition of carbonate to hydroxyapatite can occur at both the OH-group and the phosphate group^25,29^. Depending on the molecular structure and external conditions, CHAs can form crystal or amorphous micro-particles^24,30,31^ and bind various toxic chemicals^27,32,33^, including some types of porphyrins^34,35^. Therefore, one can assume that CHAs and heme in gastrointestinal tract of an animal form insoluble integrated complexes due to strong bonds between phosphate and/or carbonate groups of CHA and heme molecules. Our hypothesis is that this process occurs in chyme during its transition from the stomach to the bowels. In the stomach, under the action of digestive enzymes and high acidity (pH ~ 2)^36^, the fragments of muscles and bones are broken down with the release of heme and the dissolution of calcium phosphates and carbonates. Moving from stomach to small intestine with a neutral juice (pH ~ 6)^36^, phosphates and carbonates become insoluble and form CHA particles binding the free heme. Such encapsulation inside the CHA particles makes heme insoluble in physiological fluids and, accordingly, biologically inert. The resulting particles are excreted from the body as part of faeces.

To verify this assumption, we simulated the formation of such particles in vitro using model compounds—synthetic CHA and hemin.

### Modelling of CHA-heme complex formation

**Figure 3:**
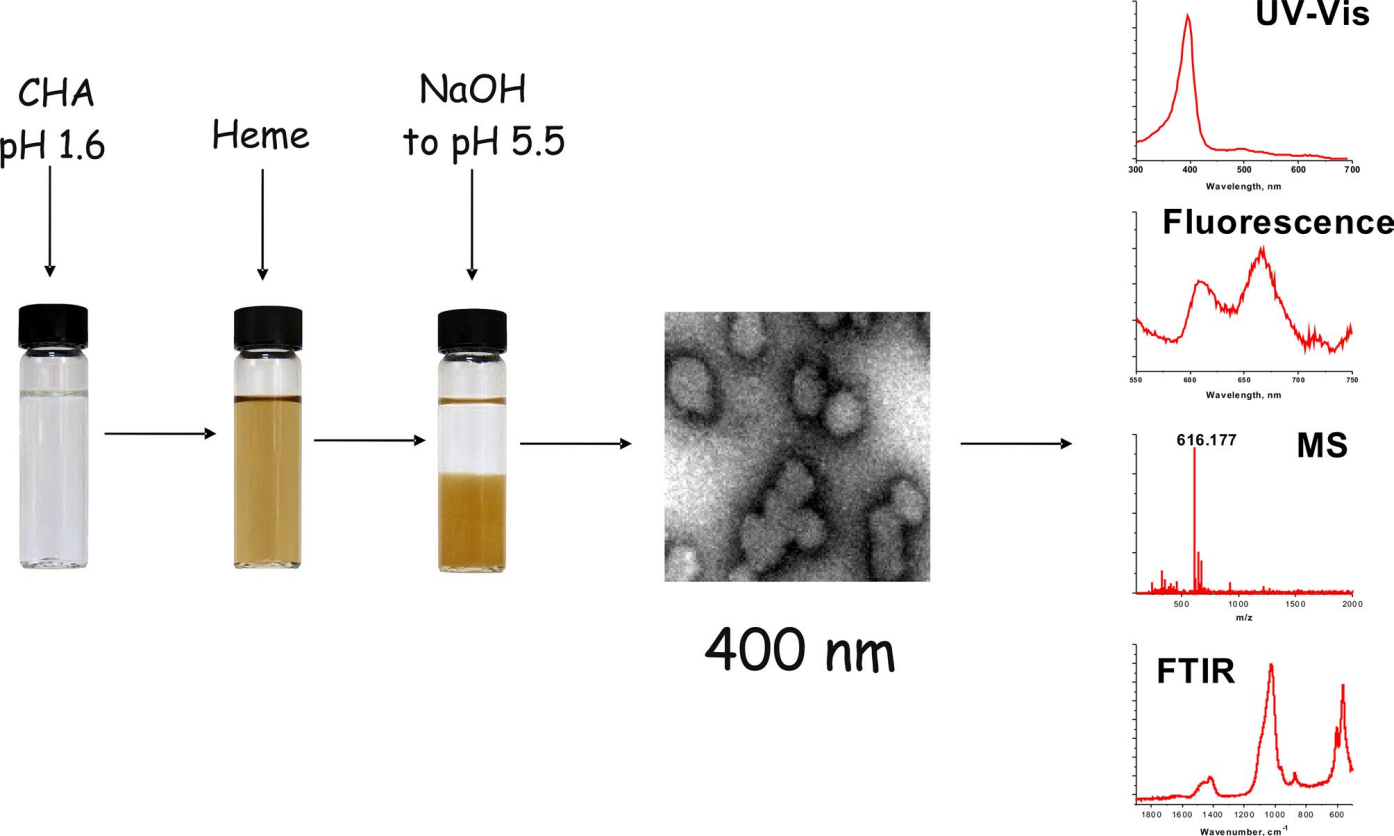
In vitro simulation of CHA-heme particles formation using synthetic CHA and hemin. A solution of CHA in dilute hydrochloric acid (pH < 2 as in the stomach) was mixed with aliquot of hemin and the resulting mixture was neutralized to pH ~ 6 (as in small intestine) by the addition of NaOH. An analysis of the formed precipitate showed that it consists of nanoparticles 50–70 nm in size. FTIR spectrum of particles was typical to that of CHA. UV–Vis absorption, fluorescence, and LC–UV–MS data of the precipitate solution in MeHCl testify the presence of heme in the formed particles.

0.5 mL of 0.1 mM hemin solution in methanol was added to 10 mL of 2.7 mg mL^−1^ CHA aqueous solution containing 0.1 M HCl (pH 1.6–1.9). The neutralization of this solution by an aliquot of NaOH to pH 5.5–5.9 resulted in the formation of amorphous brownish precipitate (Fig. 3) very similar to the one observed during the neutralization of MHEM from the cat faeces. It is important to emphasize that almost all hemin molecules (more than 95%) were co-precipitated with CHA. TEM showed that the precipitate consists of particles with 50–70 nm in size. FTIR spectrum of this material has absorption features typical to CHA (Fig. 2). The heme-containing precipitate, similar to the MHEM precipitate, was insoluble in water and methanol, but completely dissolved in MeHCl. The solution has shown the absorbance and fluorescence spectra very similar to the spectra of hemin and MHEM from cat faeces; the presence of heme was confirmed by LC–UV–MS (Fig. 3, Supplementary Fig. S3). Thus, in vitro modelling demonstrates that heme-containing nanoparticles similar to natural ones could be fabricated from heme and hydroxyapatite. This confirms the hypothesis of in vivo formation of heme-containing CHA particles.

### Observation of natural CHA particles with the use of electron microscope

Direct observation of CHA-heme particles from natural material would be undeniable evidence for the proposed mechanism of heme detoxification. To this end, the MHEM precipitate from cat faeces (MB diet) as well as the synthetic CHA-heme complexes were subjected to TEM (Supplementary Fig. S4). Round particles with broad distribution of 10–50 nm and an average size of 15–20 nm were detected in the MHEM precipitate. Though the size of synthetic CHA-heme particles was somewhat larger (grains with 50–70 nm in diameter as mentioned above), this confirms the in vitro formation of both synthetic and natural CHA-heme complexes in the form of nanosized particles.

The next step was to detect the native particles directly in cat faeces, without any preliminary dissolution of material with MeHCl. The aqueous suspension of faeces was passed through a filter of 10 × 10 µm cells, washed with water (for removing urobilin) and methanol (for removing free heme), and then dried. The yield of this filtrate was 70–80% (by dry weight) of the MHEM content in the original faeces. The TEM analysis revealed the presence of particles with 15–20 nm in size similar to the particles observed in the MHEM precipitate from cat faeces. The FTIR spectrum of dry filtrate (Supplementary Fig. S5) is almost identical to the one of CHA. Differences in shapes of bands corresponding to phosphate salts between the MHEM precipitate and the filtered native CHA-heme particles should be assigned to the maturity of apatites in native particles. The FTIR spectrum of native CHA-heme particles contains also three distinct peaks at 1650, 1550 and 1250 cm^−1^ usually related to bone collagen amides I, II, and III^30,37,38^. The presence of these peaks indicates the biogenic origin of these native CHA particles.

**Figure 4:**
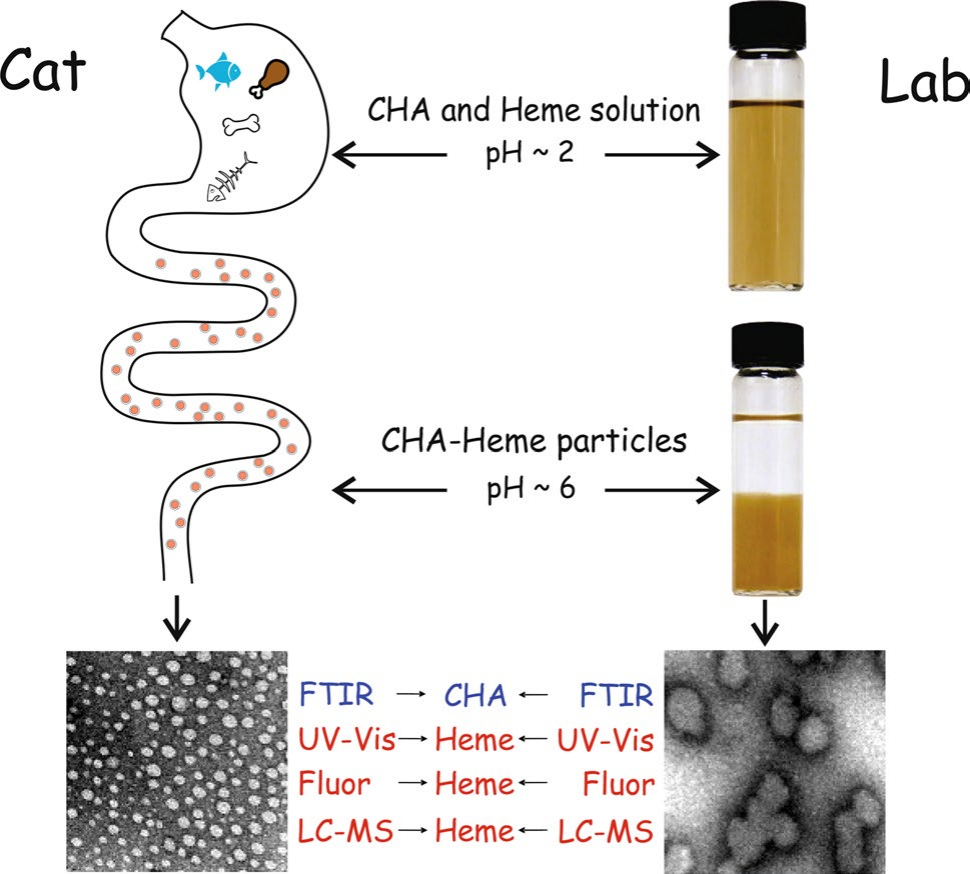
Schematic presentation of the similarity of the proposed scenario of the formation of CHA-heme particles in the gastrointestinal tract of a cat and the synthesis of similar particles in laboratory conditions. Side length of the TEM photos corresponds to 400 nm.

Heme presence in the native particles was confirmed by above-mentioned treatment and analysis by UV–Vis spectroscopy and LC–UV–MS. These results demonstrate the similarity of morphological and chemical properties of MHEM precipitate particles, native particles obtained by filtration from cat faeces, and synthetic CHA-heme particles (Fig. 4).

### Influence of cat diet on the formation of CHA-heme particles

**Figure 5:**
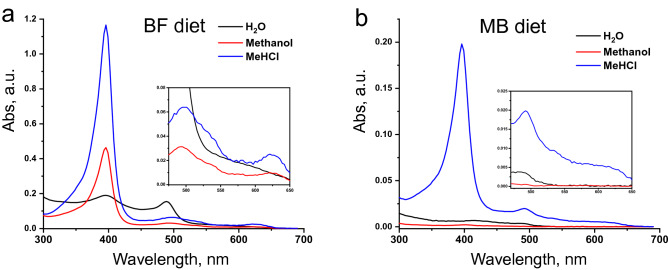
Absorption spectra of extracts obtained by sequential extraction of cat faeces (1 mg mL^−1^) with H_2_O, methanol, and MeHCl. Cat received (**a**) boneless beef meat (BF) nutrition or (**b**) chicken meat-and-bone (MB) nutrition. The heme abundance in the MHEM fraction of the BF group cats is much higher in comparison with the MB group (bound heme, blue line). The water-soluble fraction of faeces from the BF group contains higher amounts of free urobilin (black line), and the methanol-soluble fraction of faeces contains significantly higher level of free heme (red line).

**Figure 6:**
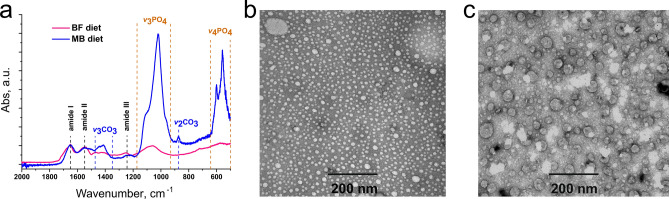
FTIR spectra (**a**) and TEM of native CHA-heme particles excreted by cat received (**b**) MB and (**c**) BF diet. It can be seen that differences in the diet significantly affect the FTIR spectra of the particles, especially the peaks of orthophosphates, whose intensity in the MB group is almost an order of magnitude higher than in the BF group. The size of particles also differ, particles formed in cats of the MB group are smaller and more uniform (15–20 nm), compared with particles of the BF group (15–70 nm).

In a natural cat diet, red meat is the main source of heme and bones are the main sources of CHA. Assuming that CHA-heme complexes are formed inside the gastrointestinal tract, one can expect that this process would depend on the animal’s diet. To verify this assumption, we studied faeces from cats that were fed bone-free red beef meat for 3 weeks (BF group). Six samples of domestic cats faeces were taken (two samples from cats “S” and “L” and one sample from cats “T” and “M”), analysed according to the described procedures and compared with the faeces data of the same cats that received meat-and-bone diet (MB group) earlier. Data comparison for the BF and the MB groups revealed the following differences and similarities (Figs. 5, 6; Supplementary Fig. S6):

A. An observation of cat defecation for the period of research showed that the amount of faeces (dry weight) excreted daily by cats by the MB group (about 4–6 g) was greater than that of the BF group (0.6–0.8 g). To compare the average daily amounts of MHEM excreted by both groups of cats, MHEM was extracted from faeces samples and precipitated. Precipitates were dried to constant weight and recalculated accordingly to the total weight of the original faeces samples. The average daily amounts of MHEM in cat faeces of the MB diet group were 462 ± 225 mg (data spread from 145 to 835 mg), and in the BF group 74 ± 18 mg (data spread from 50 to 98 mg) (Supplementary Fig. S6a).
B. The water-soluble fraction of faeces from the BF group contained significantly higher amounts of urobilin (Fig. 5; Supplementary Fig. S7) (average daily amounts 1.3 ± 0.8 mg) as compared to the MB group (0.04 ± 0.03 mg) (Supplementary Fig. S6b,c).
C. The methanol-soluble fraction of faeces from the BF group contained significantly higher level of free heme as compared to the MB group (Fig. 5; Supplementary Fig. S8). The daily content was about 0.4–2.6 mg with the average value of 1.3 ± 1.2 mg. The same fraction of the MB group contained minor amounts of heme (about 5 µg) (Supplementary Fig. S6b,c).
D. Heme containing complexes (bound heme) were found in MHEM fractions of faeces from both groups with significantly higher heme abundance in the BF group (Fig. 5; Supplementary Fig. S9). Daily bound heme content in MHEM of cats from the BF group was estimated as 2.7–7.1 mg, with the average 5.7 ± 2.0 mg. In the MB group, daily average bound heme level was 1.6 ± 0.5 mg with data ranging from 0.9 to 2.4 mg (Supplementary Fig. S6b,c).
E. Particles obtained after the filtration of faeces from the BF group exhibited broader variation in size, 15–70 nm as compared to the MB group (15–20 nm) (Fig. 6). FTIR spectra of the particles also differed: in the MB group, the intensities of phosphate signals were 6–10 times higher than that of the carbonate signals, while in the BF group these signals were of approximately equal intensities. This indicates very high degree of particles carbonization in the BF group.

We can roughly estimate the efficiency of the mechanism of heme encapsulation. The cats from the MB group received 90 g of chicken breast and 5 g of beef liver daily. The cats from the BF group received 120 g of beef meat daily. Assuming that 100 g of liver, chicken breast and beef meat contains 0.8 mg, 0.12 mg, and 0.9 mg of heme iron, respectively^39,40^, their daily consumption of heme can be estimated as approximately 1.7 mg (MB diet) and 12.2 mg (BF diet). At the same time, the daily level of bound heme in faeces was 1.6 ± 0.5 mg (MB diet) and 5.7 ± 2.0 mg (BF diet). Thus, one can estimate that a major part of heme consumed by cats from the MB group and a significant part of heme consumed by cats from the BF group has been encapsulated in the CHA-heme complexes.

Even in high-heme and bone-free animal diet most of heme was detoxified either by its decomposition down to urobilin, or by encapsulation into CHA-heme complexes. However, the presence of free heme in the methanol-soluble fraction indicates the incompleteness of heme detoxification for the BF diet.

The results of the present work strongly suggest the formation of insoluble CHA-heme complexes in the gastrointestinal tract of an animal. We assume that this process occurs in chyme during its transition from the stomach to the bowels (Fig. 4). Encapsulation inside the CHA particles is a previously unknown alternative mechanism of heme detoxification. It operates along with the well-studied enzymatic reactions of heme oxidation and decomposition down to stercobilin and urobilin. If animal food contains high levels of heme and low levels of calcium phosphates and carbonates, heme detoxification may become incomplete, and chemically active free heme is present in the animal’s faeces. This may increase the risk of developing gastrointestinal disorders. Even in the absence of bones in a cat’s diet, highly carbonated CHA-heme particles were found in cat faeces. Possibly, when there are no orthophosphates in the food, the main building material for particles are carbonates formed in the stomach and small intestine^41^. Nevertheless, even in such a “non-optimal composition”, these particles can bind and neutralize significant amounts of heme.

## Conclusion

This work demonstrates the existence of a previously unknown way of heme detoxification in the body of a domestic cat, *Felis silvestris catus*. Similar to blood-sucking invertebrates, this way is based on the conservation of toxic free heme within biologically inert crystalline structures, but mechanisms and products of these processes are completely different. We found that in the gastrointestinal tract of a cat, on the way from the stomach to the bowel, heme is included in water-insoluble CHA particles. The structure and composition of these grains, as well as the efficiency of heme inclusion, depend on animal nutrition, especially on having bones as part of the diet, a source of orthophosphate and carbonate salts. This fact suggested that with having bones in a diet, the major part of heme is encapsulated within CHA particles at the early stages of digestion without significant contribution of usual catabolic mechanisms of heme degradation into urobilin and other derivatives. Therefore, chewing on the bones, so loved by predators, is not as much of an act of enjoying the delicacy, but rather an act of collecting an important material necessary for maintaining good health.

## Methods

### Chemicals

Type I bovine hemin (chloroferriprotoporphyrin IX) and formic acid were obtained from Sigma-Aldrich (USA). Hydroxyapatite and glacial acetic acid was purchased from BDH Chemical Ltd (England). DMSO (99% purity) was purchased from Merck (Germany). Analytical grade methanol was purchased from Fisher Scientific (USA) and acetonitrile HPLC grade was purchased from Panreac (Spain). H_2_O was deionized using Ultra Clear UV plus TM water system (SG water, Germany) to the quality of 18.2 MOhm.

### Animals

The study was conducted in accordance with the European Union Directive 2010/63/EU on the protection of animals used for scientific purposes, and with the ethical approval from International Tomography Center SB RAS. No special permission from the national or local authorities is required.

Faeces samples were obtained from outbred cats—2 males (named “S” and “T”) and 2 females (named “L” and “M”) aged 1.5–10 years (Supplementary Figure S6d). All animals lived at home of different owners. The owners were fully aware of the goals and methods of the study and agreed to participate in it. According to the owners, all animals were healthy for at least 2 months before the start of the study, and there were no prerequisites for visits to the veterinarian in the next 2 months (for the study period). The owners did not plan to get other pets, as well as travel, change their place of residence, carry out redevelopment and repair work around the house for the period of research. All animals, depending on the experimental conditions, received two types of raw food for the 3 weeks: either meat-and-bone food (MB diet) ~ 120 g of ground chicken meat and bone (about 90 g of breast and 30 g of bones) and ~ 5 g of beef liver (as a source of additional heme), or bone-free food (BF diet) ~ 120 g of red boneless beef meat. Drinking water was available ad libitum. Faeces were collected by the owners no later than 30–40 min after defecation of the animals. Faeces samples were placed in a plastic container with a screw cap and transferred to the laboratory within an hour.

### Sample preparation

A fresh faeces sample weighing 7–10 g was cut into fragments of 0.1–0.3 g and dried in a laminar flow of air at room temperature to constant weight. The dried material was triturated in a porcelain mortar and sieved through a filter with a mesh size of 2 mm. Then the sifted material was sent to the study, and its remains were placed in a plastic container with a screw cap and stored at − 20 °C.

### Synthesis of β-hematin

β-Hematin, a synthetic analogue of hemozoin, was synthesized according to the method of Blauer et al.^20^ with minor modifications^21^. Briefly, 1 mL of 15 mg mL^−1^ hemin solution in 0.4 M NaOH was mixed with 890 μL of water and 970 μL of glacial acetic acid and incubated at 70 °C for 18–24 h. The resulting β-hematin crystals were collected by centrifugation (10,000×*g*, 10 min, 20 °C) and washed successively with deionized water, dimethyl sulfoxide and ethanol to an optical density in supernatant of A_400_ < 0.01. The precipitate was dried in vacuum over phosphorus pentoxide at room temperature to constant weight. The yield of β-hematin was about 95%.

### Synthesis of carbonated hydroxyapatite (CHA)

Synthesis of CHA was performed according to the method of Russo et al.^26^, based on simultaneous drop-wise addition of a phosphoric acid solution and a sodium hydrogen carbonate solution to stirred calcium oxide dispersion in distilled water. Once the addition has been completed, the suspension was aged for 24 h. The precipitate was washed with deionized water and dried at 40 °C in a laminar air flow to constant weight. The dried material was ground in a porcelain mortar and sieved through a filter with a mesh size of 2 mm. The powder obtained was verified as CHA by FTIR spectrum analysis, using published FTIR spectra of CHA and commercial hydroxyapatite as references.

### Isolation of native particles consisting of heme and CHA

500 mg of dry faeces was suspended in 40 mL of 0.05% SDS and placed in an Amicon 8050 Ultrafiltration Stirred Cell filter module (Millipore, USA). The original filter of Amicon 8050 unit was replaced with a nylon filter from a chromatographic column K50 (Pharmacia, Sweden) with mesh size 10 × 10 µm, to increase the cutoff approximately to 10 µm. The cell was placed on the magnetic stirring table, connected to a regulated nitrogen pressure source and set pressure ~ 2.5 kg cm^−2^. The filtrate with particles passing through the filter was centrifuged (10,000×*g*, 10 min, 20 °C). The precipitated particles were washed twice with deionised water, three times with methanol and dried at 40 °C in a laminar air flow to constant weight. The powder obtained was verified as CHA particles by FTIR spectrometry and TEM. To analyse the presence of heme, the particles were dissolved in MeHCl and the solution was centrifuged (10,000×*g*, 10 min, 20 °C). UV–Vis absorption spectra and LC–UV–MS data of the supernatant verified the presence of heme.

### UV–Vis spectroscopy

UV–visible electronic absorption spectra were recorded with a Cary 100 (Varian, USA) spectrophotometer and an Agilent 8453 (Agilent Technologies, USA) spectrophotometer. All UV–Vis measurements were carried out in a 10 × 10 mm quartz cell. The heme concentration was calculated by molar absorption coefficients under alkaline conditions (ε_385_ = 5.8 × 10^4^ M^−1^ cm^−1^)^42^ or under acidified methanol (MeHCl) conditions (ε_397_ = 3 × 10^5^ M^−1^ cm^−1^, measured in this work with commercially available heme). Urobilin concentration was calculated by molar absorption coefficient (ε_492_ = 6 × 10^4^ M^−1^ cm^−1^)^43^.

### Fluorescence measurements

Fluorescence spectra were recorded with FLSP920 spectrofluorometer (Edinburgh Instruments, UK). Spectra from samples containing 1 μM heme in MeHCl were obtained by summation of four independent measurements. All fluorescence spectra were obtained with a 10 × 10 mm quartz cell and corrected for the wavelength-dependent sensitivity of the detection.

### IR spectroscopy

The IR absorption spectra of the samples were recorded in the range of 4000–500 cm^−1^ (mid-IR) at room temperature using FTIR spectrometer Vertex 80v (Bruker Optics, Germany) equipped with a single reflection diamond ATR accessory A225/Q (Bruker Optics, Germany). The spectral resolution was 1 cm^−1^. Each spectrum was obtained by summation of thirty two independent measurements.

### LC–UV–MS

The HPLC separation was performed at the Center of Collective Use “Mass spectrometric investigations” SB RAS on an UltiMate 3000RS chromatograph (Dionex, Germany) using analytical column Agilent Zorbax XDB-C18 (4.6 × 250 mm, 80 Å, 5 μm). The chromatograph was equipped with a flow-cell diode array UV–Vis detector (DAD) with 190–800 nm spectral range. Solvent A consisted of 0.1% formic acid solution in H_2_O, solvent B consisted of 0.1% formic acid solution in acetonitrile. The separation was performed in isocratic mode, 75% of Solvent B; the flow rate was 1.5 mL min^−1^. The sample injection volume was 10 µL. After the DAD cell a homemade flow splitter (1:10) directed the lesser flow to an ESI-q-TOF electrospray high-resolution mass spectrometer maXis 4G (Bruker Daltonics, Germany). The mass spectra were recorded in a positive mode with 100–2000 m/z range with 1 Hz rate.

### Transmission electron microscopy (TEM)

Transmission electron microscopy was performed at the Common Use Centre for Microscopy of Biological Subjects, Institute of Cytology and Genetics SB RAS. The specimens were examined and photographed in a JEOL 1400 electron microscope (JEOL, Japan) at 100 kV. A sample deposited on a formvar coated grid and negative stained with 2% uranyl acetate^44^.

### Statistical analysis and graphical presentation

A Fisher–Pitman permutation test was performed to check the differences in the means of the MB and the BF samples on Stata statistical software with *permtest* package (StataCorp LLC, United States). For the graphical presentation of the data obtained, we used Origin 8.5.1 software (OriginLab, United States).

## Supplementary Information

Supplementary Information 1.

## Data Availability

All data presented in this study are available from the corresponding author upon request. A reporting summary for this article is available as a Supplementary Information file.
