# Supplementary material for: A previously unknown way of heme detoxification in the digestive tract of cats
Source: Sci Rep. 2021 Apr 15;11:8290. doi: 10.1038/s41598-021-87421-6 (PMC8050217; doi:10.1038/s41598-021-87421-6)
Supplement: Supplementary file 1 — Supplementary Information 1. [file 41598_2021_87421_MOESM1_ESM.doc]

**Supplementary Information**

For

**A previously unknown way of heme detoxification in the digestive tract of cats**

by Alexandr B. Duzhak^1,*^, Petr S. Sherin^2,3^, Vadim V. Yanshole^1,4^, Sergey L. Veber^5^, Sergey I. Baiborodin^6^, Olga I. Sinitsyna^4,7^, Yuri P. Tsentalovich^1^

^1^ Laboratory of Proteomics and Metabolomics, International Tomography Center SB RAS, Institutskaya str. 3a, Novosibirsk, 630090, Russia

^2^ Group of Photoinduced Processes, International Tomography Center SB RAS, Institutskaya str. 3a, Novosibirsk, 630090, Russia

^3^ Chemistry Department, Imperial College London, Molecular Sciences Research Hub, White City Campus, 80 Wood Lane, W12 0BZ, London, UK.

^4^ Novosibirsk State University, Pirogova str. 2, Novosibirsk, 630090, Russia

^5^ Laboratory of Magnetic Resonance, International Tomography Center SB RAS, Institutskaya str. 3a, Novosibirsk, 630090, Russia

^6^ **Common Use** Centre for Microscopy of Biological Subjects, Institute of Cytology and Genetics SB RAS, Acad. Lavrentiev Avenue 10, Novosibirsk, 630090, Russia

^7^ **Laboratory of Gene Engineering**, Institute of Cytology and Genetics SB RAS, Acad. Lavrentiev Avenue 10, Novosibirsk, 630090, Russia

^*^Correspondence and requests for materials should be addressed to Corresponding Author:

Alexandr B. Duzhak. email: [abduzhak@mail.ru](mailto:abduzhak@mail.ru), tel.: +73833303136

**Table of content:**

Supplementary figures

References

**Supplementary figures**

**Supplementary Fig. 1** Synthesized ß-hematin and its solubility in comparison with hemin.

(**a**) FTIR spectrum of synthesized ß-hematin. Characteristic peaks at wavenumbers 1712, 1664 and 1210 cm^-1^ [1–3] confirm the accurate β-hematin formation. (**b**) UV-Vis spectra of hemin solutions in H_2_O, methanol, 50 mM NaOH, and MeHCl. Solutions were prepared by dissolving hemin (estimated concentration of 3 μM) in the appropriate solvents with stirring for 40 min in the absence of light. The resulting solutions were centrifuged (10000 × g, 10 min), and absorption spectra were measured. The dissolved hemin had a characteristic spectrum with a Soret band at 398 nm and Q bands at 450-650 nm (inset). (**c**) UV-Vis spectra of β-hematin solutions were prepared and analysed according to the described procedure. Both hemin and hematin are soluble in 50 mM NaOH and MeHCl but insoluble in water. But unlike hemin, β-hematin is insoluble in methanol.

**Supplementary Fig. 2** Absorption spectra of extracts obtained by sequential extraction of cat faeces (1.5 mg/mL) with H_2_O, methanol, 50 mM NaOH, and MeHCl. Cat received meat-and-bone nutrition. Water and methanol extracts did not contain significant amounts of recognized compounds. NaOH extract contained only urobilin, which indirectly indicated the absence of hemozoin in the faeces of the cat. MeHCl extracted material has the absorption spectrum with Soret band (397 nm) and four Q bands (an enlarged view of the Q-bands is shown in the inset), characteristic to that of heme.

**
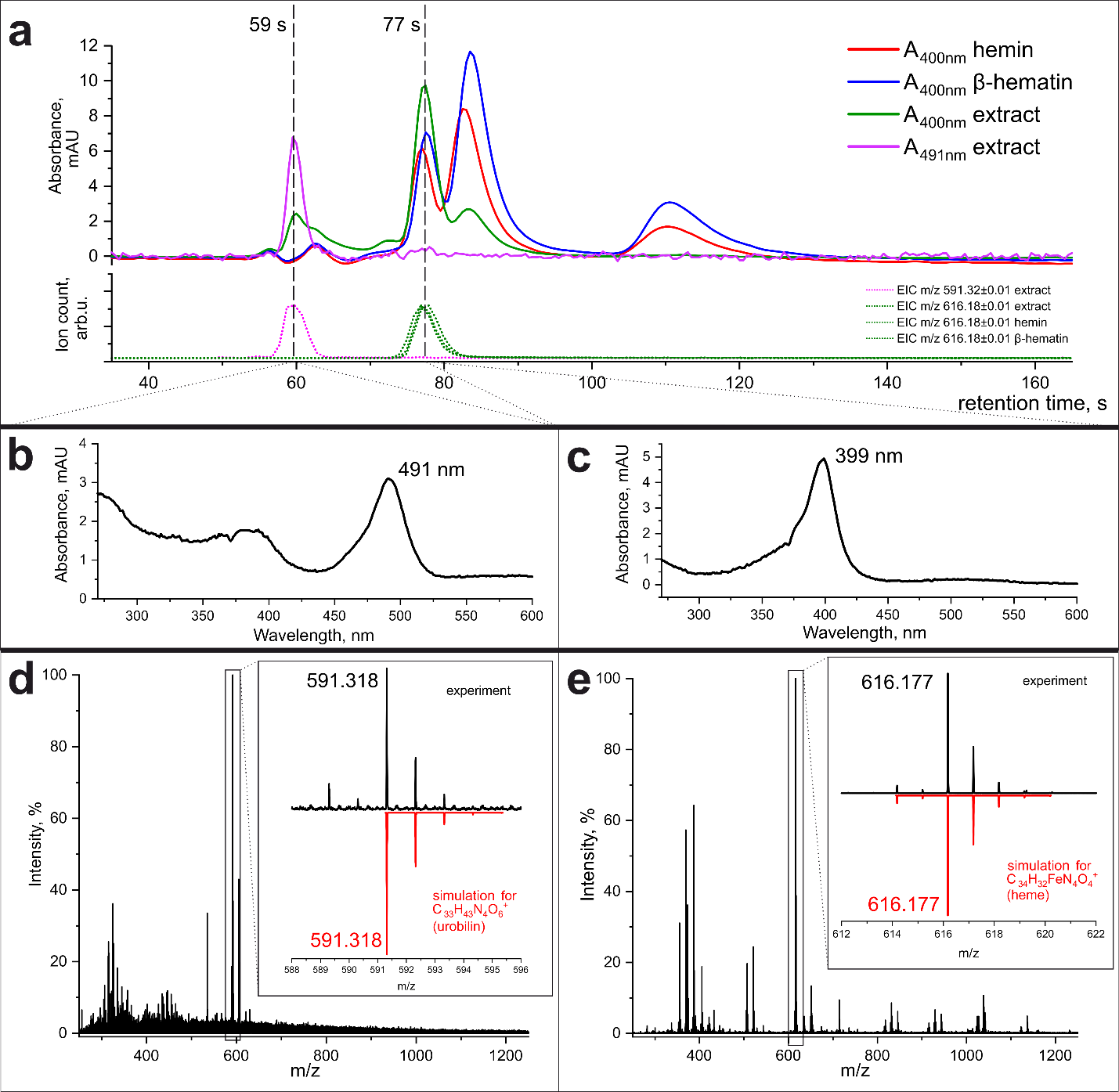
**

**Supplementary Fig. 3** LC-UV-MS analysis of MeHCl extract from the cat faeces (MB diet), hemin and β–hematin standards.

(**a**) UV chromatograms of extract monitored at 400 and 491 nm and chromatograms of hemin and β–hematin standards monitored at 400 nm; (**b**) and (**c**) UV-Vis spectra of peaks at RTs = 59 and 77 s in extract from the cat faeces; (**d**) Mass spectrum of urobilin-containing peak (RT = 59 s), inset: zoomed m/z range of experimental mass spectrum and the simulation of isotopic pattern for urobilin (C_33_H_43_N_4_O_6_^+^) in inverse scale; (**e**) Mass spectrum of heme-containing peak (RT = 77 s), inset: zoomed m/z range of experimental mass spectrum and the simulation of isotopic pattern for heme (C_34_H_32_FeN_4_O_4_^+^) in inverse scale. Mass spectra of hemin and β–hematin standards are the same (data not shown). All samples also contained heme-like molecules formed by the addition of one or two CH_2_ groups to heme (data not shown): found at RTs = 83 s and 110 s, with m/z 630.192 and 644.208, UV_max_ at 399 nm (C_35_H_34_FeN_4_O_4_^+^, theoretical m/z 630.192 and C_36_H_36_FeN_4_O_4_^+^, theoretical m/z 644.208). This addition probably took place during the sample preparation.


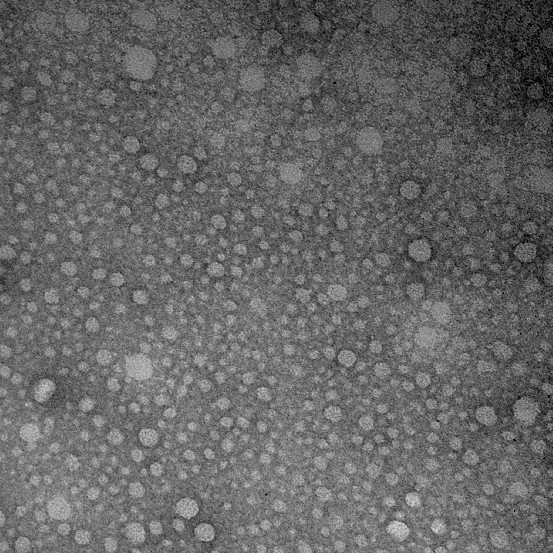

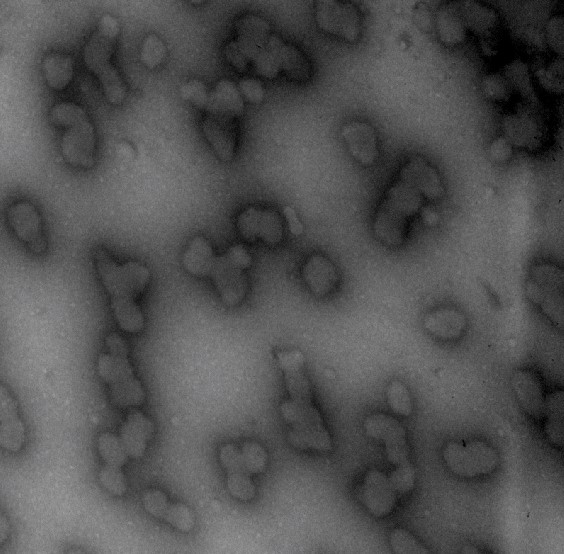


**Supplementary Fig. 4** TEM images of natural and synthetic CHA-heme particles. (**a**) Precipitated MHEM particles from faeces of cats (MB diet) form round particles with broad distribution in size of 10-50 nm and an average size of 15-20 nm. (**b**) Synthetic CHA-heme particles with the size of 50-70 nm in diameter.

**Supplementary Fig. 5** FTIR spectra of (**1**) native CHA-heme particles and (**2**) precipitated particles of MHEM from cat faeces (MB diet). FTIR spectrum of synthetic CHA (**3**) presented as reference. In contrast to the smoothed spectrum of the MHEM precipitate, natural CHA-heme particles spectrum presents absorbance bands of orthophosphate and carbonate components very similar to those of the CHA reference. Particles spectra also contain a peaks I, II and III at 1651, 1550, and 1230 cm^-1^, which are absent in the CHA spectrum. This peaks are usually related to amides I, II and III, common for the peptides and proteins [4–6].


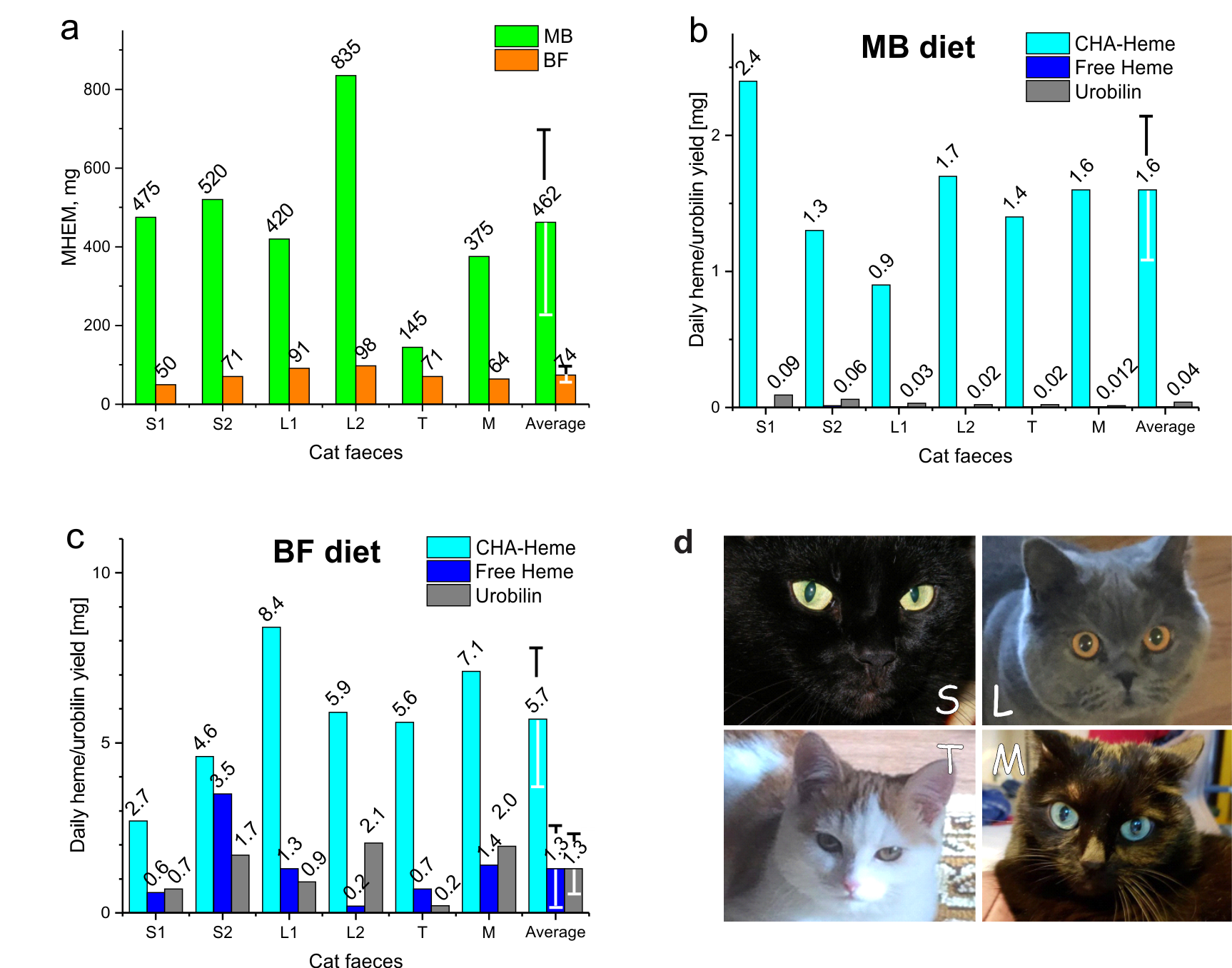


**Supplementary Fig. 6** The effect of bone-free (BF) or meat-and-bone (MB) diet on daily excretion of (**a**) MHEM, and (**b, c**) free heme, CHA-heme, and urobilin. (**d**) S, L, T, and M – our furry and unselfish collaborators. Due to small sample size, we resorted to non-parametric tests. We performed a Fisher-Pitman permutation test to check for differences in the means of MB and BF samples. The data presented show that for MB and BF diets all studied parameters (output of MHEM, CHA-heme, free heme, and water-soluble urobilin) differ statistically significantly (p = 0.002). There is substantial variation in the parameters we study, likely due to limited sample size (n=6) and the fact that the composition of faeces could be influenced by inevitable variation of quantity and composition of the animal’s diets. Rather than performing a full statistical analysis, we view these results as an illustration of how a cat’s diet influences the yield of СНА particles and their effectiveness of binding the free heme.


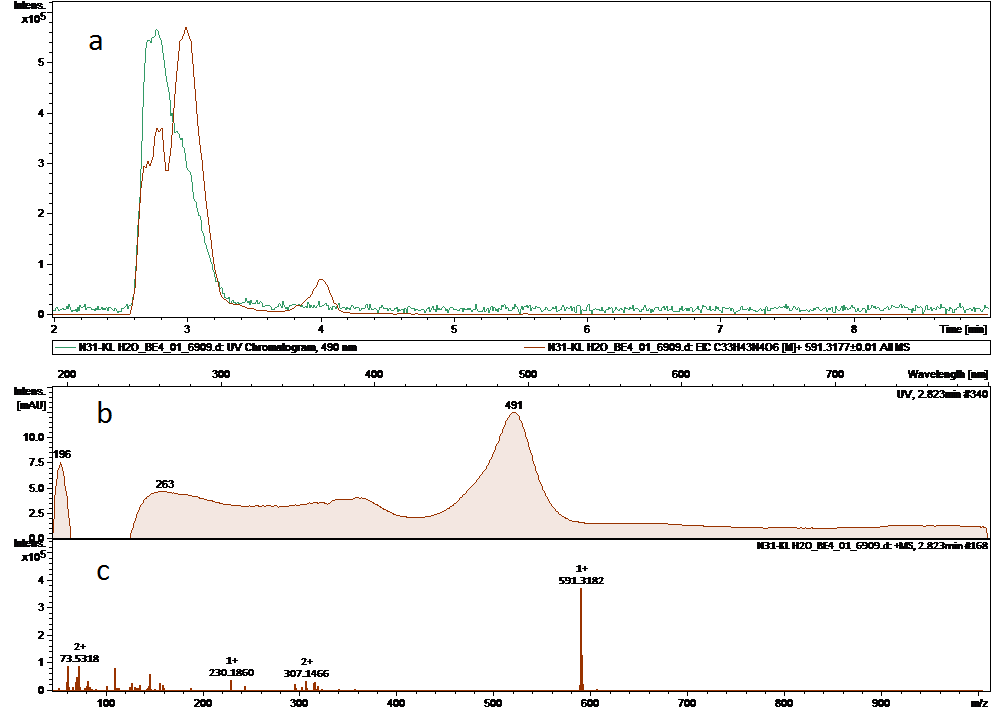


**Supplementary Fig. 7** LC-UV-MS analysis of H_2_O extract from the cat faeces (BF diet). (**a**) Optical and MS chromatograms of H_2_O extract monitored at 491 nm (green line) and m/z 591.318 (brown line); (**b**) UV-Vis spectrum of peak at RT = 2.83 min. Spectrum has the absorption with maximum at 491 nm, characteristic to that of urobilin; (**c**) Mass spectrum of urobilin-containing peak (RT = 2.83 min), with m/z 591.318 (C_33_H_43_N_4_O_6_^+^).


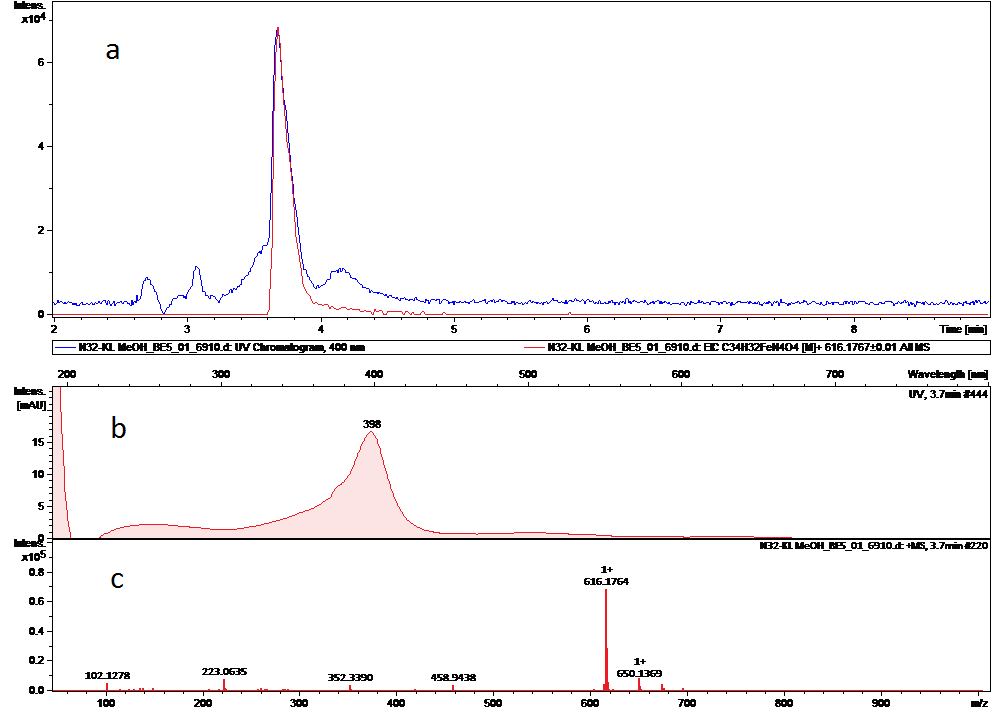


**Supplementary Fig. 8** LC-UV-MS analysis as evidence of free heme in methanol extract from the cat faeces (BF diet). (**a**) Optical and MS chromatograms of methanol extract monitored at 400 nm (blue line) and m/z 616.176 (red line); (**b**) UV-Vis spectrum of peak at RT = 3.7 min with the Soret band (398 nm) characteristic to that of heme; (**c**) Mass spectrum of heme-containing peak (RT = 3.7 min) with m/z 616.176 (C_34_H_32_FeN_4_O_4_^+^).


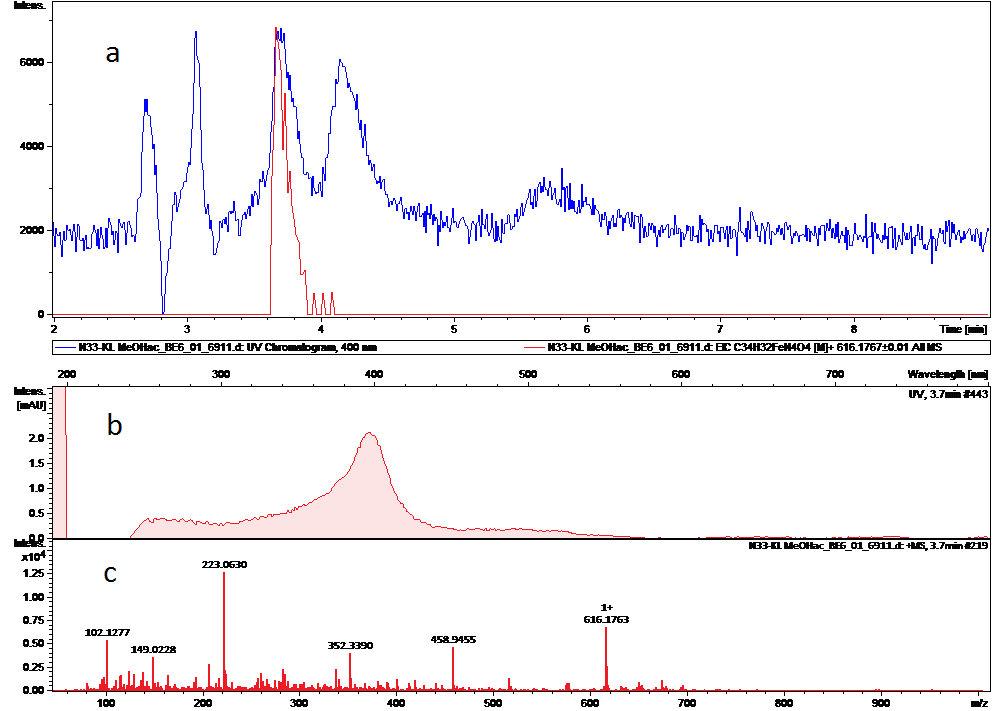


**Supplementary Fig. 9** LC-UV-MS analysis as evidence of heme in MHEM from the cat faeces (BF diet). (**a**) Optical and MS chromatograms of MHEM monitored at 400 nm (blue line) and m/z 616.176 (red line); (**b**) UV-Vis spectrum of peak at RT = 3.7 min with the Soret band (398 nm) characteristic to that of heme; (**c**) Mass spectrum of heme-containing peak (RT = 3.7 min) with m/z 616.176 (C_34_H_32_FeN_4_O_4_^+^).

**References**

1. Blauer, G. & Akkawi, M. On the preparation of β-haematin. *Biochem. J.* **346**, 249–250 (2000).

2. Jaramillo, M. *et al.* Synthetic Plasmodium-like hemozoin activates the immune response: A morphology - Function study. *PLoS One* **4**, e6957 (2009).

3. Slater, A. F. G. *et al.* An iron-carboxylate bond links the heme units of malaria pigment. *Proc. Natl. Acad. Sci. U. S. A.* **88**, 325–329 (1991).

4. Querido, W. *et al.* Fourier transform infrared spectroscopy of developing bone mineral: From amorphous precursor to mature crystal. *Analyst* **145**, 764–776 (2020).

5. Boskey, A. & Pleshko Camacho, N. FT-IR imaging of native and tissue-engineered bone and cartilage. *Biomaterials* **28**, 2465–2478 (2007).

6. De Campos Vidal, B. & Mello, M. L. S. Collagen type I amide I band infrared spectroscopy. *Micron* **42**, 283–289 (2011).
